# The Roles of GDF-9, BMP-15, BMP-4 and EMMPRIN in Folliculogenesis and In Vitro Fertilization

**DOI:** 10.3390/jcm13133775

**Published:** 2024-06-27

**Authors:** Serafeim Fountas, Efthymia Petinaki, Stamatis Bolaris, Magdalini Kargakou, Stefanos Dafopoulos, Athanasios Zikopoulos, Efthalia Moustakli, Sotirios Sotiriou, Konstantinos Dafopoulos

**Affiliations:** 1 Fertility and Sterility Unit, Elena Venizelou General-Maternity District Hospital, 11521 Athens, Greece; serfountas@gmail.com (S.F.); sbolaris@gmail.com (S.B.); magda.kargakou@yahoo.gr (M.K.); 2 Department of Microbiology, University Hospital of Larissa, 41110 Larissa, Greece; petinaki@uth.gr; 3 Department of Health Sciences, European University Cyprus, 2404 Nicosia, Cyprus; sn191957@students.euc.ac.cy; 4 Royal Devon and Exeter Hospital, Barrack Rd, Exeter EX 25 DW, UK; thanzik92@gmail.com; 5 Laboratory of Medical Genetics, Faculty of Medicine, School of Health Sciences, University of Ioannina, 45110 Ioannina, Greece; thaleiamoustakli@gmail.com; 6 Department of Embryology, Faculty of Medicine, School of Health Sciences, University of Thessaly, 41110 Larissa, Greece; sotiriousoti@yahoo.gr; 7 ART Unit, Department of Obstetrics and Gynecology, Faculty of Medicine, School of Health Sciences, University of Thessaly, 41110 Larissa, Greece

**Keywords:** GDF-9, BMP-4, BMP-15, EMMPRIN, normal follicular development, folliculogenesis, in vitro fertilization

## Abstract

Growth differentiation factor 9 (GDF-9) contributes to early ovarian development and oocyte survival. Higher concentrations of GDF-9 in follicular fluid (FF) are associated with oocyte nuclear maturation and optimal embryo development. In in vitro fertilization (IVF), GDF-9 affects the ability of the oocyte to fertilize and subsequent embryonic development. Bone morphogenetic protein 15 (BMP-15) is involved in the regulation of ovarian function and affects oocyte development. During IVF, BMP-15 contributes to the formation of competent blastocysts. BMP-15 may play a role in embryo implantation by affecting endometrial receptivity. Bone morphogenetic protein 4 (BMP-4) is involved in the regulation of follicle growth and development and affects granulosa cell (GC) differentiation. In relation to IVF, BMP-4 is important for embryonic development, influences cell fate and differentiation, and plays a role in facilitating embryo–endometrial interactions during the implantation process. Extracellular matrix metalloproteinase inducer (EMMPRIN) is associated with ovulation and follicle rupture, promotes the release of mature eggs, and affects the modification of the extracellular matrix of the follicular environment. In IVF, EMMPRIN is involved in embryo implantation by modulating the adhesive properties of endometrial cells and promotes trophoblastic invasion, which is essential for pregnancy to occur. The purpose of the current article is to review the studies and recent findings of GDF-9, BMP-15, BMP-4 and EMMPRIN as fundamental factors in normal follicular development and in vitro fertilization.

## 1. Introduction

During a normal pregnancy, there are significant physiological adaptations occurring in the uterus, placenta, and maternal circulatory system. The pregnant uterus undergoes growth and expansion to accommodate the developing fetus adequately. Concurrently, the placenta and helical arteries undergo alterations to ensure an ample blood and nutrient supply to support fetal growth. Additionally, notable hemodynamic changes occur in maternal circulation, including increased heart rate, expanded plasma volume and cardiac output, and decreased vascular resistance. These adjustments are orchestrated to optimize blood flow to various organs and reduce strain on the maternal heart. The structural remodeling and functional adjustments in utero-placental tissue and maternal circulation associated with pregnancy represent crucial aspects of gestational physiology [1,2].

The process of embryo selection remains a pivotal consideration in in vitro fertilization (IVF) cycles, particularly in light of legislation mandating single embryo transfer in many countries [3]. The quality of the embryo is intricately linked to the quality of the oocyte, highlighting the significance of oocyte assessment [4]. Traditional approaches have relied on morphological parameters to evaluate oocyte quality; however, studies examining the correlation between oocyte morphology and subsequent embryo quality have yielded conflicting findings, prompting a reevaluation of this paradigm [5,6,7,8,9,10]. Consequently, recent investigations have shifted focus towards exploring factors within the follicular fluid (FF) that may influence oocyte developmental potential. FF comprises a complex milieu of plasma components, blood-derived factors, and secretory products from oocytes, granulosa cells, and sheath cells [11].

The influence of the follicular microenvironment on oocyte development and embryo quality has been a subject of extensive research since the 1990s [12]. Both oocyte and embryo quality are intricately affected by the hormonal and growth factors present in FF, emphasizing their significant role [13]. However, the precise mechanisms through which these molecules operate remain incompletely understood.

Among the growth factors present in FF, bone morphogenetic proteins (BMPs) and growth differentiation factor 9 (GDF-9), members of the transforming growth factor-β (TGF-β) superfamily, play pivotal roles in oocyte and follicle maturation [14,15] and contribute to various aspects of ovarian function and follicle development, and this is the main reason these proteins were chosen to be reviewed. In particular, growth differentiation factor 9 (GDF-9), bone morphogenetic protein 15 (BMP-15), bone morphogenetic protein 4 (BMP-4) and extracellular matrix metalloproteinase inducer (EMMPRIN) proteins are the subjects of discussion and analysis due to their pivotal roles in ovarian function and female fertility. GDF-9 is particularly essential in the early stages of follicle development, being secreted by oocytes and exerting influence on the proliferation and differentiation of granulosa cells, which are imperative for the establishment of a healthy follicle. Additionally, GDF-9 facilitates communication between the oocyte and surrounding somatic cells, thereby directly impacting oocyte quality and maturation, crucial for successful fertilization and subsequent embryo development. Furthermore, BMP-15 often collaborates with GDF-9 in regulating folliculogenesis, thereby enhancing the efficiency of these processes and underscoring the critical nature of both proteins in proper follicular development [16]. BMP-15 also plays a significant role in regulating granulosa cell function, providing essential support and signaling during oocyte maturation [17]. On the other hand, BMP-4 is involved in the initial phases of follicle development, contributing to cell survival and differentiation, which prepares the follicle for subsequent maturation stages. Moreover, BMP-4 influences the ovarian microenvironment by acting on various somatic cells within the ovary, ensuring an environment conducive to follicle development [18]. Lastly, EMMPRIN is indispensable for extracellular matrix (ECM) remodeling, a process vital for follicle expansion and ovulation. By inducing matrix metalloproteinases (MMPs), EMMPRIN ensures dynamic ECM remodeling to support follicular development. Additionally, it activates intracellular signaling pathways such as MAPK and PI3K/AKT, promoting cell proliferation, survival, and differentiation, thereby further enhancing follicular development and ovulation [19,20].

Mutations impacting the function of these proteins have been associated with decreased fertility in humans, hindering successful conception [21]. The oocyte itself contributes significantly to follicular growth and development through the secretion of its own growth factors, which act paracrinely on the granulosa cells. Mutations in the GDF-9 gene have been identified as causal factors in fertility or infertility in various species, including mice, sheep, and humans. Notably, women with premature ovarian failure (POF) [22,23,24] and mothers of polyfollicular dizygotic twins [25] have been found to carry numerous GDF-9 mutations [26].

GDF-9 and BMP-15 predominantly govern granulosa cell interaction and oocyte quality, while BMP-4 plays a fundamental role in the initiation of follicle formation. EMMPRIN, on the other hand, orchestrates the structural remodeling necessary for follicle expansion and subsequent ovulation. The proper functioning of these proteins is paramount for female fertility, as disruptions in their pathways can lead to infertility or other reproductive disorders, underscoring their significance as targets for fertility research and therapeutic interventions. These proteins are subjects of extensive study aimed at comprehensively understanding reproductive biology and devising effective treatments for infertility. Their involvement in cell signaling, extracellular matrix remodeling, and follicular development renders them central to discussions regarding ovarian physiology and fertility treatments. The collective analysis of GDF-9, BMP-15, BMP-4, and EMMPRIN offers a holistic perspective on the molecular mechanisms governing ovarian function and female fertility, thereby aiding in the development of advanced reproductive technologies and therapeutic strategies to address infertility [16,17,18,19,20].

Moreover, hormones, particularly those involved in the hypothalamic–pituitary–ovarian (HPO) axis, regulate the entire process of oogenesis. Gonadotropin-releasing hormone (GnRH) from the hypothalamus stimulates the release of FSH and luteinizing hormone from the pituitary gland. FSH promotes follicular development and stimulates the granulosa cells to produce estrogen, which is essential for the growth and maturation of the oocyte. LH triggers ovulation and stimulates the production of progesterone by the corpus luteum, which prepares the uterus for implantation [27]. Additionally, other hormones such as anti-Müllerian hormone (AMH) and inhibin also play roles in regulating folliculogenesis and oocyte maturation. AMH inhibits primordial follicle recruitment, while inhibin suppresses FSH secretion, thereby regulating follicular development and ovulation [28,29]. Age is another crucial factor that affects oogenesis. In females, the number of primordial follicles, which contain immature oocytes, declines with age due to a process called ovarian follicle atresia. This reduction in the ovarian reserve leads to a decline in fertility and an increased risk of chromosomal abnormalities in oocytes, such as aneuploidy. Additionally, aging is associated with changes in hormonal levels, including a decrease in the levels of estrogen and inhibin, which further impact follicular development and ovulation [30]. Therefore, both hormones and age are intricately involved in the process of oogenesis, influencing follicular development, oocyte maturation, and ultimately female fertility.

## 2. Relevant Sections

### 2.1. Growth Differentiation Factor (GDF-9)

GDF-9 is a member of the TGF-β family and plays a critical role in the early development of the ovaries and the survival of oocytes. It stimulates the proliferation of granulosa cells, facilitates follicle maturation, and contributes to oocyte formation. High levels of mature form of GDF-9 in FF are closely associated with oocyte nuclear maturation and embryo quality of development [21]. Oocyte maturation requires a combination of nuclear and cytoplasmic maturation. Evaluating nuclear maturation is generally considered simpler and more precise than assessing cytoplasmic maturation. Notably, a significant correlation has been identified between nuclear maturation and levels of mature GDF-9, offering strong evidence for the involvement of this paracrine factor in oocyte development [31]. The absence of the GDF-9 gene impedes follicular development to advanced stages, resulting in female infertility. Interestingly, concurrent loss of inhibin, an ovarian hormone, in GDF-9 mutants allows the follicles to resume growth and development [32].

Folliculogenesis is regulated by growth factors produced by oocytes, with numerous growth factors, including GDF-9, identified in mammalian oocytes to date [33]. GDF-9 acts as a paracrine factor within the TGF-β family, facilitating intracellular communication between oocytes and somatic cells, such as granulosa cells, from the early primary follicle stage through to ovulation [34,35].

While the quality of the oocyte is crucial in determining the quality of the embryo, the mechanisms underlying GDF-9 activity are particularly noteworthy. GDF-9 is secreted by the oocyte as an inactive precursor protein and undergoes proteolytic cleavage to form a mature dimer, which then becomes biologically active [36,37]. GDF-9 subsequently binds to type I and type II serine/threonine kinase receptors on granulosa cells, typically involving BMPR-II (type II receptor) and ALK5/ALK6 (type I receptors). Upon ligand binding, a series of events occur: the type II receptor phosphorylates the type I receptor, activating its kinase domain. The activated type I receptor then phosphorylates SMAD2/3 proteins, leading to their activation. Phosphorylated SMAD2/3 forms a complex with SMAD4, which translocates to the nucleus. This complex regulates the transcription of target genes involved in cell proliferation, differentiation, and survival [38,39,40,41,42].

GDF-9 performs various functions, including promoting the proliferation and differentiation of granulosa cells, preventing apoptosis, and supporting cholesterol biosynthesis in cumulus cells [43,44,45].

Under in vivo conditions, GDF-9 has been shown to stimulate cumulus cell development, inducing the production of a hyaluronic acid-rich matrix during cumulus cell expansion [46]. Moreover, GDF-9 is implicated in the proliferation of granulosa cells in antral follicles before ovulation [47,48]. Studies have reported that recombinant GDF-9 promotes the growth, development, and survival of human follicles, and the injection of exogenous GDF-9 into mouse oocyte maturation culture medium enhances embryo viability and subsequent development [43,49].

An in vivo investigation revealed that the growth factor GDF-9 plays a central role in regulating sheath cell functions. Intraperitoneal injections of GDF-9 in immature rats facilitated the progression of primordial and primary follicles into small preovulatory follicles and led to an upregulation of CYP17 expression in sheath cells [50]. Additionally, GDF-9 stimulates testosterone production in rat preantral follicle cultures. The androgen receptor antagonist flutamide blocks GDF-9-stimulated preantral development, suggesting that GDF-9 acts on both theca cells and granulosa cells to induce the development of preantral follicles at the early antral stage [51].

In the context of in vitro fertilization, GDF-9 plays a crucial role in acquiring oocyte competence and significantly influences the oocyte’s ability to fertilize, subsequently impacting embryonic development. GDF-9 is actively engaged in essential follicle–oocyte interactions (maturation, fertilization, embryonic development) required for successful fertilization, affecting both in vitro oocyte maturation and embryo viability [43]. It is necessary for dynamic processes and communication that occur between the oocyte and the surrounding cells and environment as it undergoes maturation and becomes a mature oocyte. These interactions are crucial for successful oocyte development, ovulation, fertilization, and subsequent embryonic development. This role is substantiated by experiments demonstrating that the introduction of exogenous GDF-9 to FSH-cultured mouse granulosa cell–oocyte complexes, prior to in vitro fertilization and subsequent transfer to recipient dams, did not alter the implantation rate or the weight of fetal and placental tissues. However, it did lead to an increase in the number of live embryos [43]. 

Alterations in the in vitro culture microenvironment can also significantly impact embryonic development. Recent advances have focused on leveraging cumulus granulosa cells and growth factors to enhance the developmental potential of oocytes [52,53]. Zhu et al. (2008) [54] demonstrated that co-culturing immature oocytes with cumulus cells increased the likelihood of polar body exclusion by up to 10% within a 24 h period; however, the embryogenesis rate in these oocytes remained relatively low. Previous studies have investigated the influence of growth factors on embryonic development, with GDF-9 being a key factor secreted by human oocytes during primary follicular development [55]. GDF-9 affects both the early and late stages of follicle growth, primarily by inducing granulosa cell division, suppressing follicle-stimulating hormone (FSH) receptor expression and inducing kit-ligand (KitL) expression in granulosa cells [56,57,58]. Additionally, GDF-9 induces inhibin-β production and exerts paracrine control over inhibin production in granulosa cells [59].

In vitro studies have shown that GDF-9 can sustain survival, stimulate both oocyte and follicle growth, and activate primordial follicles when cultured in situ. This has been demonstrated with ovarian tissue fragments from women [49] and goats [60]. When preantral follicles were cultured in isolation, GDF-9 effectively promoted the growth of follicles and oocytes in mice [61]. Positive outcomes were observed when a medium containing ascorbic acid was supplemented with 200 ng/mL GDF-9, enhancing the survival of isolated preantral follicles cultured in vitro, particularly in goats [62,63]. However, there is currently a lack of information on the effect of GDF-9 on the in vitro culture of isolated early antral follicles in mammals.

Research on the expression of GDF-9 and its correlation with infertility in individuals with endometriosis is limited. Observations indicate lower GDF-9 expression in women with endometriosis. Recent studies on the granulosa cells of women with endometriosis undergoing controlled ovarian stimulation revealed a significant reduction in GDF-9 mRNA expression in those with moderate to severe endometriosis compared to women without the condition. This reduction results in compromised oocyte maturation and embryo quality [64,65,66]. GDF-9 levels were found to be lower in the endometriosis group than in the control group, although a direct relationship between individual GDF-9 levels and oocyte quality was not established [67].

The ovarian phenotype characterized by cystic formations is similar to the ovarian morphology observed in women with polycystic ovary syndrome (PCOS). PCOS is the most prevalent cause of anovulation, infertility, and menstrual cycle irregularities in women, affecting approximately 5–10% of women of reproductive age [68]. Analysis of ovarian tissues from women with PCOS revealed a notable delay and decrease in GDF-9 expression during the growth and differentiation phases [69]. In a study comparing oocytes collected from women with and without PCOS after ovarian stimulation, these oocytes gradually matured through in vitro maturation. The results indicated dynamic changes in GDF-9 mRNA expression in the oocytes of women without PCOS [70]. Specifically, the expression of GDF-9 decreased from the germinal vesicle (GV) stage to the metaphase I (MI) stage, contrary to findings by Chen et al. (2009) [71]. Subsequently, GDF-9 expression increased from the MI to the metaphase II (MII) stage, reaching its peak at the final mature stage. In contrast, oocytes from women with PCOS showed no statistically significant changes in GDF-9 expression, and the expression levels in mature oocytes did not reach those observed in normal oocytes.

### 2.2. Bone Morphogenetic Protein (BMP-15)

BMP-15, a member of the TGF-β family, is crucial in regulating ovarian function and influencing oocyte development. It plays a significant role in controlling ovulation rates and oocyte quality, thereby contributing to the initiation of pregnancy [72]. Additionally, BMP-15 actively promotes follicular growth and exerts significant influence during the initial gonadotrophin-independent phases of folliculogenesis. It regulates the production of follicular granulosa cells, protecting these cells from apoptosis and enhancing their proficiency in supporting oocyte maturation [73]. The involvement of BMP-15 in early follicular development exhibits species-specific characteristics, highlighting distinctions between mono- and poly-ovulatory species [74,75]. Within ovarian follicle development, the BMP-15 gene plays a crucial role in the proliferation and differentiation of granulosa cells [76]. It encourages granulosa cell division while concurrently suppressing the expression of FSH receptors. Due to the significant impact of BMP-15 gene mutations on ovulation rate, litter size, and fertility, this gene is recognized as a key player and a potential marker for breeding in livestock, particularly in sheep [77,78]. BMP-15 exhibits the characteristic cysteine motif found in TGF-β factors, comprising a specific sequence of cysteine amino acids. Notably, BMP-15 diverges from most of these factors by possessing only six of the seven cysteine residues [34,35,79]. Functionally, BMP-15 acts as a potential activator of cell mitosis, contributing significantly to granulosa cell proliferation [31]. As an X-linked gene, its expression spans from the primary stage of oocytes to ovulation [80]. The BMP-15 gene is crucial for promoting granulosa cell growth and regulating folliculogenesis, particularly during the FSH-dependent phase [81]. 

Researchers have investigated the potential involvement of the BMP-15 gene in premature ovarian insufficiency (POI) [82,83]. The BMP-15 protein consists of an *n*-terminal pro-domain and a C-terminal mature domain. During the assembly of the BMP-15 molecule, the pro-domain folds to form a mature growth factor dimer [84]. A furinase-like protease, released from the oocyte, subsequently cleaves the BMP-15 protein into several fragments. Unlike many other factors, BMP-15 typically functions as a non-covalent dimer due to the absence of cysteine residues necessary for forming intermolecular disulfide bonds [85]. The rearrangement of the pro-domain allows human BMP-15 (hBMP-15) to be expressed as a type I (ALK6) and type II (BMPRII) receptor group [86]. BMP-15 significantly stimulates mRNA expression of granulosa KitL, a crucial factor in early follicular development [85]. In this capacity, BMP-15 acts as a negative regulator in the interaction between the oocyte and somatic granulosa cells. It promotes the expression of KitL by granulosa cells, which in turn inhibits BMP-15 expression from the oocyte through the binding of KitL to its receptor on the oocyte surface [31,73]. Moreover, BMP-15 is initially secreted as an inactive precursor by the oocyte and subsequently undergoes proteolytic cleavage to form a mature, active dimer. Additionally, BMP-15 binds to both type I and type II serine/threonine kinase receptors located on granulosa cells [41]. Common receptors in this process include BMPR-II (type II receptor) and ALK6/ALK4 (type I receptors). Furthermore, upon ligand binding, the type II receptor phosphorylates the type I receptor, which in turn phosphorylates SMAD1/5/8 proteins. Ultimately, phosphorylated SMAD1/5/8 proteins form a complex with SMAD4, which translocates to the nucleus to regulate the transcription of genes involved in granulosa cell proliferation and differentiation [33,34,82,87,88,89,90].

Throughout the development of ovarian follicles, BMP-15, secreted by the oocyte, significantly contributes to follicle development through the paracrine signaling pathway [91]. Its involvement extends from the progression of primordial to primary follicles to the inhibition of early luteinization [92]. BMP-15 exerts regulatory control over the steroidogenesis acute regulatory protein (StAR) and hinders early follicular luteinization by suppressing the luteinizing hormone receptor (LHR). These effects on follicle cell function can ultimately result in the absence of dominant follicles [93]. Following ovulation, the rapid reduction of these oocyte factors in the corpus luteum amplifies StAR expression, thereby regulating follicle maturation by modulating the differentiating effects of FSH [58]. Additionally, BMP-15 has a significant role in governing ovulation rates, oocyte health, and the initiation of pregnancy [72]. 

In vitro studies on murine granulosa cells reveal that BMP-15 stimulates granulosa cell proliferation and KitL expression while concurrently inhibiting FSH receptor expression [94]. Furthermore, in vitro fertilization studies highlight the significance of BMP-15 in embryonic development, contributing to the formation of competent blastocysts. BMP-15 may also influence embryo implantation by affecting endometrial receptivity. Given the consistent expression of BMP-15 by the oocyte throughout all stages of follicular development, including post-ovulation from cumulus–oocyte complexes, BMP-15 levels in FF are considered predictive of oocyte quality and the subsequent quality and development of corresponding embryos. Research demonstrates a statistically significant association between higher levels of BMP-15 in FF and elevated fertilization rates, increased early hatching rates, and improved quality of embryos with potentially normal development. Additionally, studies reveal a statistically significant positive correlation between BMP-15 levels in the FF of women with a poor response to ovarian stimulation programs. In this context, higher levels of BMP-15 are linked to an improved response in women, manifested by an increased number of produced follicles and, consequently, a heightened rate of fertilization and pregnancy [78,95]. A significant challenge in the in vitro growth of ovarian preantral follicles is the lower rates of oocyte maturation and embryo development compared to in vivo samples. Recognizing this, and in light of various researchers’ efforts to enhance follicle development rates in diverse culture systems using different factors, a study was conducted to assess follicular growth and development in mouse ovaries under the influence of BMP-15 [96].

Furthermore, maintaining female fertility fundamentally relies on the normal functioning of BMP-15 in the ovary. Research in sheep has uncovered various mutations in the BMP-15 gene, which correlate with increased folliculogenesis rates in heterozygotes and infertility in homozygotes [21,97,98]. Similarly, human studies underscore the crucial role of BMP-15 in preserving female fertility. Infertile women experiencing early ovarian failure syndrome and PCOS have been found to carry BMP-15 gene mutations [82,83,94]. However, insights from studies involving BMP-15 gene knockout mice suggest that the regulation of folliculogenesis and ovulation does not follow a uniform pattern across species. Homozygous BMP-15-null mice exhibit normal follicular development but experience mild infertility, primarily due to minor dysfunctions in ovulation and fertilization [94]. Among the BMPs, BMP-15 is particularly important for enhancing reproductive performance in female livestock [99]. Its physiological effects are especially pronounced during the early stages of follicle development in sheep [91] and are evident across various livestock species during this stage [58,100]. BMP-15 influences the ovulation rate in livestock through various mechanisms. It actively participates in ovarian follicle formation, and its impact is modulated by the binding protein follistatin, which binds to BMP-15 and blocks its effect on FSH receptor expression in granulosa cells [74,101]. As more follicular lesions synthesize luteinizing hormone receptor (LHR), additional follicles continue developing in the pre-ovulatory phase even as follicle-stimulating hormone receptor (FSHR) levels decrease, resulting in increased oocyte secretion during the anovulatory phase [102]. Mutations in the BMP-15 gene in domestic animals have been linked to higher ovulation rates and a greater proportion of twins in heterozygous animals, while homozygous animals exhibit infertility [74]. This mutation leads to elevated FSHR and LHR levels, increasing granulosa cell density, potentially inhibiting granulosa cell apoptosis, and enhancing ovulation rates [100,101,102]. Reduced BMP-15 signaling sensitizes granulosa cells to FSH, promoting follicle selection during the follicular phase and leading to improved ovulation in mutant females (ewes) [103]. Conversely, homozygous females (ewes) with BMP-15 mutations exhibit early inhibition of follicle growth and reduced granulosa cell sensitivity to FSH due to inhibited FSH receptor expression [102]. In mutant sheep and cattle with low BMP-15 levels, BMP-15 helps control granulosa cell proliferation by suppressing FSH receptor expression [103,104,105]. Throughout follicle and oocyte maturation in farm animals, BMP signaling significantly impacts fertility by regulating sex hormone secretion, gonadotropin receptor gene expression, and oocyte quality [58,104]. Additionally, BMP signaling is closely associated with ovulation rates and the estrous cycle [15].

### 2.3. Bone Morphogenetic Protein (BMP-4)

Ovarian stromal and cumulus cells are known to synthesize BMP-4, which is purported to exert a pivotal role in facilitating the transition from primordial to primary follicles [35]. This protein has been associated with the augmentation of primary and secondary follicles, as well as an increased abundance of primary follicles [106]. As a constituent of the TGF-β family, BMP-4 is intricately involved in gene regulation, exerting influence over gene expression within the oocyte. It is initially synthesized as an inactive precursor protein by various cell types within the ovarian environment. Following proteolytic cleavage, BMP-4 undergoes activation, leading to the formation of a mature dimer [28]. Additionally, BMP-4 interacts with type I and type II serine/threonine kinase receptors present on ovarian somatic cells, including granulosa and theca cells. Key receptors involved in this interaction include BMPR-II (type II receptor) and ALK3/ALK6 (type I receptors). Upon ligand binding, the type II receptor phosphorylates the type I receptor, initiating downstream signaling cascades. Subsequently, the activated type I receptor phosphorylates SMAD1/5/8 proteins, resulting in the formation of a complex with SMAD4. This complex translocates to the nucleus, where it regulates gene transcription processes crucial for early follicular development, cellular survival, and differentiation [33,42,82,88,89,90].

During the course of typical follicle development, BMP-4 assumes a regulatory role in orchestrating the growth and maturation of follicles, thereby influencing the differentiation of granulosa cells. In the realm of IVF, BMP-4 holds considerable significance for embryonic development, exerting influence over cellular destiny and participating in facilitating interactions between the embryo and the endometrium during the implantation process. The precise mechanistic underpinnings of BMP-4 in the follicle remain incompletely elucidated. Nevertheless, it is hypothesized that BMP-4 likely impedes the apoptosis of human granulosa cells, retards early differentiation, and preserves their steroidogenic properties [107]. In a conducted study [21], increased levels of BMP-4 were noted in women who achieved clinical pregnancy compared to those who did not, verifying the existing literature on the pivotal role of BMP-4 in follicular development. Notably, the inactivation of BMP-4 leads to embryonic lethality, attributed to abnormalities both within and outside the embryo, including aberrations in the formation of the posterior/ventral mesoderm [108].

The significance of BMP-4 in the intricate process of folliculogenesis is notable. Its presence has been consistently detected in the ovaries of female mice throughout all stages of follicular development, indicating a potential involvement in the transition from primordial to primary follicles [109]. Furthermore, BMP-4 is implicated in the regulation of both oocytes and granulosa cells [110,111]. In vitro investigations have revealed a substantial augmentation in cell numbers in oocytes treated with BMP-4 in a dose-dependent manner, with this response being hindered in a dose–response relationship when co-administered with noggin. Administration of BMP-4 into rat ovaries resulted in a notable increase in developing primary follicles and an associated decrease in primordial follicles. Additionally, it was observed that the inclusion of neutralizing BMP-4 antibodies in the culture medium led to diminished ovary size, reduced oocyte and primary follicle counts, and increased cellular apoptosis [112]. Treatment with 100 ng/mL BMP-4 for 6 days in the in vitro culture media has been associated with an enlargement of primary and secondary follicles. However, some animal studies have contested the impact of BMP-4 on the transition process from primordial to primary follicles [14]. Nevertheless, the loss of BMP-4 function leads to the impairment of the formation of primordial germ cells, which serve as the embryonic precursors of gametes (spermatocytes or oocytes) [79]. Furthermore, BMP-4 has been detected in various ovarian compartments, including the ovarian surface epithelium, corpus luteum, and around follicle stromal cells [111,113,114]. Notably, BMP-4 exhibits strong expression in oocyte cumulus cells. Moreover, the influence of BMP-4 on GCs induces substantial alterations in the response of FSH on estradiol and progesterone production in mammalian ovaries [115].

### 2.4. Extracellular Matrix Metalloproteinase Inducer (EMMPRIN)

EMMPRIN, also recognized as Basigin (CD147), is a transmembrane glycoprotein belonging to the immunoglobulin superfamily [116]. Initially denoted as tumor cell-derived collagenase-stimulating factor (TCSF), it underwent renaming to EMMPRIN due to its involvement in inducing extracellular matrix metalloproteinases via both normal and pathological cell–cell interactions [117]. Its ramifications span across tissue remodeling and various pathological conditions such as cancer, rheumatoid arthritis, heart failure, and atherosclerosis. EMMPRIN serves to stimulate MMP-9 production and can modulate MMPs in endothelial cells and tumors [118,119]. A plethora of studies underscore EMMPRIN’s involvement in processes including angiogenesis, neuronal signaling, tumorigenesis, wound healing, follicle maturation, and embryo implantation [120,121,122,123,124].

Throughout the course of normal follicle development, EMMPRIN plays a pivotal role in ovulation and follicular rupture, facilitating the release of mature eggs. It exerts influence over the modification of the extracellular matrix within the follicular environment, a critical factor for the proper development of follicles. In the context of a healthy full-term pregnancy, EMMPRIN contributes to maintaining the delicate balance between uterine contraction and relaxation, a regulation pivotal for normal pregnancy. Any disruption in these mechanisms may precipitate preterm labor, with adverse consequences for both the pregnancy and the prematurely born infant. Initial research has explored the levels of EMMPRIN in human FF, highlighting its potential significance in reproductive processes [21]. In the context of the IVF, EMMPRIN emerges as an active participant in embryo implantation, modulating the adhesive properties of endometrial cells and facilitating trophoblastic invasion—processes crucial for successful pregnancy outcomes [21,124].

The precise physiological mechanisms underlying EMMPRIN’s involvement in follicle growth and corpus luteum formation remain elusive. Nonetheless, a prevailing hypothesis suggests that EMMPRIN facilitates the activation of matrix metalloproteinases, thereby promoting crucial processes such as follicular development, ovulation rupture, and corpus luteum formation. Studies have reported a significant decrease in fertilization rates in oocytes deficient in EMMPRIN [117]. Actually, EMMPRIN is a transmembrane glycoprotein localized on the surface of various cells within the ovary, including granulosa cells and theca cells. It stimulates the expression and activation of matrix metalloproteinases (MMPs) in neighboring cells. MMPs are enzymes responsible for degrading components of the extracellular matrix (ECM). By inducing MMP expression, EMMPRIN facilitates the remodeling of the ECM, which is essential for follicular expansion and ovulation [125,126,127]. ECM degradation enables the structural changes necessary for follicle growth and oocyte release. Furthermore, EMMPRIN has the capability to activate intracellular signaling pathways, such as the MAPK and PI3K/AKT pathways. These pathways play crucial roles in cell proliferation, survival, and differentiation [128,129,130]. Of crucial importance is the noted decline in placental MMP expression during pregnancy, which mirrors a corresponding reduction in placental EMMPRIN expression. This underscores EMMPRIN’s significance as a principal stimulator of MMPs in diverse tissues throughout pregnancy. The etiology behind the observed decrease in placental MMPs and EMMPRIN in late pregnancy remains uncertain, potentially linked to the placenta’s capacity to generate MMPs or the varying roles of MMPs at different stages of pregnancy.

While EMMPRIN exhibits diverse molecular and cellular properties, its principal function resides in stimulating the synthesis of various matrix metalloproteinases [131]. EMMPRIN molecules are expressed at varying levels across epithelial, endothelial, and leukemic cells [132], as well as in a multitude of other cell types [133]. EMMPRINs are implicated in a variety of fundamental cellular processes, including lymphocyte sensitization and intracellular trafficking [134]. In the context of cancer and inflammatory disorders, EMMPRINs, either directly or indirectly through their interaction with partner molecules, can instigate the activation of matrix metalloproteinases responsible for ECM degradation, cell adhesion, and intercellular communication. Moreover, they have the capacity to induce the differentiation of myofibroblasts associated with ECM deposition and contraction. The precise mechanisms governing the transition between fibrosis and lysis remain elusive; however, EMMPRINs may contribute by up-regulating MMPs secreted by neighboring fibroblasts, thereby assuming a pivotal role in promoting tumor invasion, growth/progression, and metastasis—an assertion supported by existing evidence [124,135].

In rats, an elevation in ovarian EMMPRIN levels during luteinization has been observed, implying its functional significance in this context [16,136]. Disruptions in EMMPRIN have been linked with infertility in both male and female mice [95,109]. EMMPRIN is implicated in various reproductive processes, including oocyte maturation, fertilization, implantation, and early embryonic development [21,117,137], exerting influence over follicle regulation and the degeneration of follicular tissue. While extensive research has delineated the role of EMMPRIN in tumor growth and metastasis, its precise mechanisms in oral squamous cell carcinoma (OSCC) tumorigenesis remain incompletely understood [138]. However, in the realm of reproduction, EMMPRIN emerges as a crucial cell surface molecule involved in early embryogenesis. Experimental findings indicate its significance in development and reproduction, as evidenced by studies involving knockout mice lacking the basigin gene (Bsg), which predominantly succumb before or after implantation, with the few surviving adult mice exhibiting infertility [139]. EMMPRIN’s activity in modulating the heightened expression of matrix metalloproteinases (MMPs) induced by sex hormones underscores its role in reproductive physiology. Additionally, EMMPRIN undergoes upregulation in the uterus and aorta of pregnant rats and virgin rats treated with sex hormones [140], suggesting its involvement in hormonal regulation during pregnancy [141].

Despite observations from various animal studies indicating elevated EMMPRIN levels during follicle growth and luteinization, lower EMMPRIN levels were reported in FF samples from women achieving clinical pregnancy. This discrepancy may stem from differences in measurement methods for EMMPRIN; previous studies primarily relied on immunohistologic staining, whereas the present investigation measured EMMPRIN levels in FF samples. Alternatively, the variation could be associated with the correlation between EMMPRIN and luteinization. In a rat model, an increase in EMMPRIN mRNA levels was found during luteinization post-ovulation [136]. Notably, increased EMMPRIN expression during the early corpus luteum (CL) phase in mouse ovaries declines following human chorionic gonadotropin (hCG) stimulation [142]. If increased EMMPRIN levels indeed correlate with follicular luteinization, assessing follicular EMMPRIN levels could serve as an indicator of early luteinization in patients undergoing IVF treatment [117]. The observed reduction in fertility in EMMPRIN-null mutant oocytes, coupled with EMMPRIN’s localization in granulosa cells and theca cells during the pre-ovulatory phase, underscores its efficacy in follicle formation and oocyte maturation [117,124].

## 3. Interaction between Proteins

### 3.1. Interaction between GDF-9 and BMP-15 

GDF-9 and BMP-15, belonging to the TGF-β superfamily, are pivotal in multiple facets of typical follicle development, encompassing oocyte growth, granulosa cell function, and follicle maturation. Their significant contributions to female fertility and infertility have been extensively corroborated in scholarly investigations [37,73,81,142,143]. Both factors are indispensable for normal reproductive processes and may serve as indicators of embryo development. They govern the provision of granulosa cells to the oocyte, serve as a defense against reactive oxygen species, and furnish essential nourishment to the oocyte through mechanisms such as cholesterol biosynthesis and glycolysis [35,143,144]. 

BMP-15 contributes to granulosa cell proliferation, differentiation, and follicular development, facilitating the transition of follicles from the early to the FSH phase. Conversely, GDF-9 assumes a pivotal role in orchestrating ovarian function and enhancing the developmental competence of oocytes intrinsically. It forms non-covalent heterodimers within the oocyte, exerting synergistic effects on adjacent follicular granulosa cells. This cooperative mechanism implies that BMP-15 and GDF-9 collaborate to promote ovulation, sustain oocyte vitality, and support the occurrence of pregnancy [145]. GDF-9 and BMP-15 frequently collaborate synergistically to regulate folliculogenesis and oocyte development. These proteins are co-expressed within oocytes and exert their influence on surrounding granulosa cells. GDF-9 and BMP-15 bind to specific serine/threonine kinase receptors present on granulosa cells, thereby initiating intracellular signaling cascades. This activation results in the phosphorylation of SMAD proteins, notably SMAD2 and SMAD3, which subsequently form complexes with SMAD4. These complexes translocate to the nucleus, where they regulate the transcription of target genes essential for granulosa cell proliferation, differentiation, and follicle development. The synergistic interplay between GDF-9 and BMP-15 augments the efficiency of these processes, thereby facilitating the proper maturation of follicles and oocytes [34,42,144].

Due to their potential to forecast oocyte quality and quantity, along with their substantial positive correlation with antral follicle count, the secretion of GDF-9 and BMP-15 in FF can serve as markers of ovarian reserve. The concentrations of these factors in FF reflect the quality (maturity) of retrieved oocytes post-stimulation. Investigations have delved into the link between serum levels of BMP-15 and GDF-9 during IVF and the yield of produced oocytes, particularly in women afflicted with genital disorders and polycystic ovary syndrome [146,147,148,149]. The evident nexus between mutations/deletions in GDF-9 and BMP-15 genes and their biologically active properties in follicular cells underscores the significance of these factors in female infertility [143,144].

It has been suggested that high BMP-15 levels in FF are associated with enhanced fertilization rates and embryo development [95]. However, these disparate findings may stem from variations in experimental conditions and dimeric structures. Notably, a significant correlation was found between FF levels of mature forms of GDF-9 and BMP-15. In mice, BMP-15 was identified as a multimer consisting of the pro-region and mature protein when bound to GDF-9 [150]. Additionally, the effects of mutations in BMP-15 were only apparent when the mutant BMP-15 protein was co-expressed with GDF-9 [151]. Importantly, this effect was intensified in co-expression scenarios and aligns with the concept that GDF-9 may exert substantial biological activity against BMP-15 in humans [152].

Enhancements in the development of in vitro matured oocytes to the blastocyst stage were observed with the administration of GDF-9 or BMP-15 to oocyte complexes [153]. Furthermore, evidence from human FF supports the idea that IVF outcomes could be improved by supplementing the oocyte culture medium with the mature form of GDF-9 [154].

Dysfunction of these two factors may indicate primary ovarian failure (POF), a condition impacting ovarian function that impedes the production of mature follicles in young women [84]. In cases of GDF-9 deficiency, there is a reduction in granulosa cell proliferation, abnormal oocyte growth, and a failure of follicles to progress beyond the primary stage [16]. GDF-9 also plays a role in inhibiting granulosa cell apoptosis and preventing follicle closure. The mature forms of GDF-9 and BMP-15 can exist as homodimeric or heterodimeric structures [152]. However, the significance of proforms, mature forms, and bilaterals in humans remains uncertain. Mature forms of BMP-15 and GDF-9 were not detected in the FF of 39.5% and 4.9% of infertile women, respectively, suggesting a potential issue in the production of mature forms of these growth factors in these individuals. Possible explanations include mutations in the GDF-9 and BMP-15 genes leading to reduced protein production or incomplete post-transcriptional processing of proforms, contributing to a diminished production of mature forms. A more comprehensive analysis is necessary to elucidate the challenges associated with generating biologically active forms of these growth factors [144,153].

Conversely, BMP-15 induces mitosis and the proliferation of granulosa cells [75]. The identification of homozygous mutations in GDF-9 and BMP-15 in infertile sheep underscores their critical role in these species [154]. Mutations in BMP-15 have also been observed in women with ovaries secreting abnormal levels of gonadotropins [82]. Both GDF-9 and BMP-15 act to inhibit gonadotropin-stimulated progesterone secretion [35,155]. Additionally, these two paracrine factors secreted by oocytes regulate the proliferation, apoptosis, metabolism, and expansion of cumulus cells [37].

A significant correlation has been established between the levels of mature GDF-9 and BMP-15 in FF [156]. In conjunction with GDF-9, mouse BMP-15 is observed to form a polymer of prodomains and mature proteins [150]. Furthermore, the impact of BMP-15 mutants became evident only when the mutant BMP-15 proteins were co-expressed with GDF-9 [151,152]. These findings substantiate the proposition that GDF-9 exerts a substantial influence on BMP-15 in humans [153].

Further studies have indicated that both GDF-9 and BMP-15 influence steroidogenesis and impede premature luteinization [90,156]. Specifically, while GDF-9 alone does not stimulate steroidogenesis, its co-administration suppresses progesterone and estradiol production induced by FSH [127]. A significant discovery is the negative correlation observed between mature forms of GDF-9 and progesterone levels in FF, suggesting the involvement of this paracrine factor in the luteinization process. Therefore, the elevated levels of mature GDF-9 in FF are evidently associated with oocyte nuclear maturation and embryo development [153].

A combination of autocrine/paracrine factors play a crucial role in the growth and differentiation of ovarian follicles [157]. In domestic animals, the expression patterns of BMPs ligands and receptors suggest the significant involvement of the BMPs system and GDF-9 in this intrafollicular network [91]. The BMPs system and GDF-9 jointly regulate granulosa cell proliferation, differentiation, and apoptosis throughout follicle formation [158], acting through autocrine/paracrine mechanisms within the ovary [159]. Oocyte-derived growth factors GDF-9 and BMP-15 are selectively expressed by oocytes from the primordial stage in ruminants and collaboratively influence litter size during follicle development [160]. These oocyte growth factors are essential not only in the early stages but also in the later stages of follicle development [35]. They play a fundamental role in the differentiation of various granulosa cells, regulating crucial granulosa cell functions and influencing gene expression patterns in somatic follicle cells [145]. Additionally, BMP-15 and GDF-9 control the expression of hyaluronan synthase 2, crucial for matrix formation necessary for cumulus cell expansion, thereby participating in the stimulation of cumulus cell expansion [58]. Furthermore, both GDF-9 and BMP-15 significantly contribute to oocyte health and the occurrence of pregnancy, regulating the ovulation rate [34,72].

There is supporting evidence for a combination of BMP-15 and GDF-9 mutations, as demonstrated by females with mutations in both BMP-15 and GDF-9 ovulating more frequently compared to heterozygotes with mutations in only one of them [72]. Consequently, it becomes crucial to consider the interplay between BMP-15 and GDF-9 in follicle development, which subsequently influences the number of births in livestock.

### 3.2. Interaction between BMP-4 and EMMPRIN

The interaction between BMP-4 and EMMPRIN involves BMP-4’s influence on early follicular development and cellular survival, as well as EMMPRIN’s role in ECM remodeling by inducing MMP expression and activating intracellular signaling pathways. BMP-4 functions through serine/threonine kinase receptors and SMAD proteins, predominantly influencing early follicular development and cellular survival [34]. EMMPRIN facilitates ECM remodeling by inducing MMP expression and activating intracellular signaling pathways to support cell proliferation and differentiation, which are pivotal for follicular development and ovulation. Grasping these mechanisms is imperative for enhancing reproductive technologies and therapeutic interventions in fertility treatments [28,32,39,44].

Both BMP-4 and EMMPRIN contribute to processes associated with follicular development and pregnancy, albeit in distinct capacities, as integral components of the intricate regulatory network governing the female reproductive process. They are integral to a signaling pathway involving interactions with other growth factors, cytokines, and extracellular matrix components [21].

A correlation was identified between BMP-4 levels and clinical pregnancy, and the combination of BMP-4 with EMMPRIN demonstrated a sensitivity and specificity of 67% each in predicting clinical pregnancy [21]. Cumulative evidence suggests that the quantity of BMP-4 in FF serves as a reliable marker for predicting the outcome of IVF/ICSI. While the existing literature implies a positive impact of these cytokines on follicle maturation and implantation, it merely hints at the potential use of these markers for predicting clinical pregnancy. Consequently, the findings of the aforementioned study do not offer an interpretation of the relationship between the levels of these proteins and embryo development and quality. However, the higher rate of MII (metaphase II) oocytes in the pregnancy group supports the hypothesis that BMP-4 and EMMPRIN might play a role in follicle formation and oocyte maturation [21].

BMP-4 collaborates with EMMPRIN to facilitate follicular development and ovulation. BMP-4 is secreted by various cells within the ovary, including granulosa and theca cells. It interacts with specific serine/threonine kinase receptors on ovarian somatic cells, such as granulosa and theca cells, initiating signaling pathways that promote cell survival, differentiation, and follicular development [36,37]. Concurrently, EMMPRIN, localized on the surface of various ovarian cells, induces the expression and activation of MMPs in neighboring cells. MMPs are enzymes responsible for degrading components of the ECM. Through inducing MMP expression, EMMPRIN facilitates the remodeling of the ECM, thereby enabling structural changes necessary for follicle expansion and ovulation. Collectively, BMP-4 and EMMPRIN contribute to the dynamic regulation of the ovarian microenvironment, promoting follicular development and ovulation [38,39,40].

## 4. Conclusions

In summary, female infertility represents a significant health issue, with genetic factors playing a pivotal role. GDF-9 contributes to early ovarian development and oocyte viability, stimulates granulosa cell proliferation, facilitates follicle maturation, and participates in oocyte formation. Higher concentrations of mature GDF-9 in FF are notably associated with oocyte nuclear maturation and embryo development. BMP-15 regulates ovarian function and impacts oocyte development. During IVF, BMP-15 is essential for embryonic development and aids in the formation of competent blastocysts. BMP-4 is involved in gene regulation within the oocyte, influencing gene expression patterns. In normal follicle development, it governs follicle growth and differentiation and influences granulosa cell maturation. In the context of IVF, BMP-4 is crucial for embryonic development, modulates cell fate and differentiation, and facilitates embryo–endometrial interactions during implantation. EMMPRIN contributes to follicle regulation and degeneration of follicular tissue. During normal follicular development, it is associated with ovulation and follicle rupture, promotes the release of mature eggs, and affects modifications in the extracellular matrix of the follicular environment, which is vital for follicle development. In the context of IVF, EMMPRIN plays a role in embryo implantation by modulating the adhesive properties of endometrial cells and promoting trophoblastic invasion, essential for successful pregnancy establishment.

Conclusively, GDF-9 and BMP-15 exhibit synergistic effects on each other. Their cooperative mechanism implies that these variables collaborate to promote ovulation, sustain oocyte vitality, and support the occurrence of pregnancy. Therefore, their interaction suggests a synergistic effect, where the combined action of both GDF-9 and BMP-15 enhances the efficiency of folliculogenesis and oocyte development. Moreover, the relationship between BMP-4 and EMMPRIN appears to be more synergistic than antagonistic. Although they have distinct roles, their actions seem to complement each other in promoting follicular development and tissue remodeling. Therefore, their interaction suggests a synergistic effect, where the combined action of both BMP-4 and EMMPRIN contributes to the overall regulation of folliculogenesis and ECM dynamics.
